# Role of inflammation and oxidative stress in the etiology of primary ovarian insufficiency

**DOI:** 10.4274/tjod.00334

**Published:** 2016-09-15

**Authors:** Elif Ağaçayak, Neval Yaman Görük, Hakan Küsen, Senem Yaman Tunç, Serdar Başaranoğlu, Mehmet Sait İçen, Ahmet Yıldızbakan, Hatice Yüksel, Sevgi Kalkanlı, Talip Gül

**Affiliations:** 1 Dicle University Faculty of Medicine, Department of Obstetrics and Gynecology, Diyarbakır, Turkey; 2 Memorial Hospital, Clinic of Obstetrics and Gynecology, Diyarbakır, Turkey; 3 Şırnak State Hospital, Clinic of Obstetrics and Gynecology, Şırnak, Turkey; 4 İdil State Hospital, Clinic of Obstetrics and Gynecology, Şırnak, Turkey; 5 Dicle University Faculty of Medicine, Department of Biochemistry, Diyarbakır, Turkey; 6 Dicle University Faculty of Medicine, Department Immunology and Medical Biology-Genetic, Diyarbakır, Turkey

**Keywords:** Premature ovarian insufficiency, oxidative stress, inflammation

## Abstract

**Objective:**

The aim of this study was to elucidate the etiology and treatment of primary ovarian insufficiency, which is of unknown cause in 95% of the cases.

**Materials and Methods:**

Thirty patients aged 18-40 years who presented to Dicle University Faculty of Medicine Clinic of Obstetrics and Gynecology between June 2012 and January 2014 and were diagnosed as having primary ovarian insufficiency based on their clinical and endocrinologic data, and 30 healthy controls were included in this study.

**Results:**

No significant differences were found between patients with primary ovarian insufficiency and control subjects in demographic data and lipid profile levels, thyroid- stimulating hormone, prolactin, and glucose. However, the neutrophil to lymphocyte ratio and levels of follicle-stimulating hormone, luteinizing hormone, total antioxidant status, total oxidant status, and oxidative stress index were significantly higher in patients with primary ovarian insufficiency than in control subjects. In the correlation analysis, follicle-stimulating hormone exhibited a positive correlation with total oxidant status, oxidative stress index, and the neutrophil to lymphocyte ratio (r=0.573** p<0.001, r=0.584** p<0.001, r=0.541 p<0.001, respectively) and correlated negatively with total antioxidant status (r=-0.437** p<0.001).

**Conclusion:**

The neutrophil to lymphocyte ratio, total oxidant status, and oxidative stress index levels are elevated in primary ovarian insufficiency. Therefore, anti-oxidative and anti-inflammatory treatment might be administered to patients in the early stage of primary ovarian insufficiency. However, larger studies are needed to clarify whether these elevated levels are a cause or a consequence of primary ovarian insufficiency.

## INTRODUCTION

The age of normal menopause, which occurs as a result of the depletion of functional primordial follicles in ovaries, is 50±4 years^(1)^. Loss of normal ovarian functions before the age of 40 years is referred to as primary ovarian insufficiency (POI)^(2)^. In women under the age of 40 years who experience amenorrhea for longer than 4 months, and follicle-stimulating hormone (FSH) levels found at the menopausal range on two consecutive occasions with an interval of at least one month suggests POI^(3^). Ovaries do not cease all function in approximately 50% of affected women, and 5-10% of these women can get pregnant and give birth after the diagnosis of POI^(3,4,5)^.

POI is a common disorder that afflicts 1-2% of women younger than 40 years and 0.1% of women aged less than 30 years^(6)^. It is a heterogeneous disorder that demonstrates great variations in causes and phenotypes. The focus of the present study was the etiology of POI, which affects one out of every 100 women under the age of 40 years.

Follicle depletion is caused by insufficient formation of the primordial follicle pool in the intrauterine period, increased follicle consumption, and autoimmune or toxic follicle degradation. Follicle dysfunction, on the other hand, occurs when normal functions of ovarian follicles are blocked by a pathologic process, such as mutations of the FSH receptor^(7)^ either mechanism results in functional insufficiency of the ovary.

POI comprises various diseases with a wide variety of pathogenesis such as genetic chromosomal, mitochondrial, enzymatic, iatrogenic, and immunologic aberrations, or infections^(8)^. These causes may effect the ovary at each period of life, including prepubertal, pubertal, and reproductive periods^(9)^. However, POI in patients is generally idiopathic, and the principal mechanisms are unknown.

Oxidative stress (OS) causes lipid peroxidation, functionally and structurally. It changes protein and DNA, supports apoptosis, and conduces the risk of chronic diseases such as cancer and heart disease by the agency of effects on redox status and/or redox-sensitive signaling pathways and gene expression^(10)^. Findings from in vitro, animal model, and clinical studies proposed that OS was implicated in the etiology of reverse reproductive cases in both women and men^(11)^.

The neutrophil to lymphocyte ratio (NLR), which is an indicator of systemic inflammation, demonstrates the balance between neutrophil and lymphocyte concentrations in the body. Inflammatory cytokines induce DNA damage and inhibit DNA repair^(12)^. Studies showed that inflammatory mediators were increased in patients with premature ovarian failure (POF)^(13)^. There are many rat studies in the literature regarding anti-inflammatory treatments in the treatment of POF^(14)^. This is the first study in the literature to investigate NLR, total antioxidant status (TAS), and total oxidant status (TOS) in POI, which is of unknown cause in 95% of the cases. The purpose of this study was to elucidate the etiology and therapeutic approaches of POI.

## MATERIALS AND METHODS

A total of 30 patients aged 18-40 years of age (mean: 28.9±6.8 years) who presented to Dicle University Faculty of Medicine Clinic of Obstetrics and Gynecology between June 2012 and January 2014 and were diagnosed as having POI based on their clinical and endocrinologic data, and 30 healthy controls who were matched for ethnic background and age were included in the study. The features of 30 patients with presumptive primary ovarian insufficiency were investigated. The initial diagnosis was based on a serum FSH level higher than 40 mIU/mL in karyotypically normal women aged younger than 40 years who experienced menstrual irregularity or amenorrhea. Following the provision of written informed consent, FSH, luteinizing hormone (LH), estradiol (E2), thyroid-stimulating hormone (TSH), prolactin (PRL), triglyceride (TRG), total cholesterol (total-C), and glucose tests, as well as complete blood count were performed in the patients with POI and control subjects. Patients with thyroid diseases, hyperprolactinemia, Cushing’s disease, congenital adrenal hyperplasia or infectious diseases (pulmonary diseases, peritonsillar abscesses), cardiovascular diseases, endometrial lesions, malignant neoplasm), and patients that were administered such agents as hormonal agents, ovulation-inducing agents, glucocorticoids, anti-androgens, and anti-hypertensives over the six months prior to the study were excluded. The patients were enrolled if they were not on any drugs, had no history of recent infection or inflammation, were nonsmokers, and also had normal weight. Patients with secondary amenorrhea or pregnancy were also excluded.

In addition to family history of metabolic disorders, a detailed history including menstrual cycle pattern, temporal profile, severity of unwanted hair growth, and drug intake was taken at the time of enrollment. Weight and height measurements were made. Body mass index (BMI) was determined using the following formula: Weight (kg)/height (m^2^). One single observer performed transabdominal ultrasonography (USG) in all the participants to demonstrate ovarian morphology. To reveal possible gynecologic abnormalities, transvaginal USG was performed as appropriate using a Voluson 730 expert sonography 1.8 GHZ probe. The parameters examined in this study were as follows: Age, BMI, smoking, family history, co-existing conditions, complete blood count results, baseline hormone levels, NLR, TAS, and TOS values, and findings on sonography. This study, which was approved by the Local Ethics Committee of the university, was performed in the Obstetrics and Gynecology Clinic of Dicle University.

**Laboratory Tests**

Participants gave blood samples between the third and the fifth days of the normal menstrual cycle, i.e. in the early follicular phase. Venous blood was taken from the forearm between 08:00-10:00 AM following a fast of eight hours. Blood samples were centrifuged without delay, and sera were kept at a temperature of -80 °C before laboratory testing.

Levels of serum glucose, TRG, and total-C were determined using an Architect C 16000 autoanalyzer (Abbott Laboratories, Abbott Park, IL, USA).

FSH, LH, E2, PRL, TSH levels were determined using electrochemiluminescence immunoassay on a Cobas 601 analyzer (Roche Diagnostics, Mannheim, Germany).

For the determination of NLR, complete blood counts of patients with POI and controls were studied using a CELL-DYN automated hematology analyzer (Ruby-Abbott Diagnostics, USA).

TAS levels were measured using Erel’s^(15)^ recently developed automated technique. In Erel’s^(15)^ technique, a hydroxyl radical is generated, which is the most potent biologic radical. A solution of ferrous ion in Reagent 1 is combined with Reagent 2 that containing hydrogen peroxide in the assay. The radicals generated by the hydroxyl radical in this way are also potent radicals. As a result, the anti-oxidative action of the sample against the potent-free radical reaction is determined, which is triggered by the hydroxyl radical. The findings are reported in mmol Trolox Eq/L.

TOS levels were measured using Erel’s^(16)^ automated technique. In this technique, a ferrous ion-o-dianisidine complex is oxidized to ferric ion by oxidants found in the sample. Glycerol molecules, which are found in large amounts in the reaction medium, increase the oxidation reaction. In acidic material, the ferric ion creates a colored complex with xylenol orange. The intensity of color is measured spectrophotometrically and is related to the total amount of oxidant molecules in the sample. Calibration of the assay is performed using hydrogen peroxide. The findings are reported in µmol H_2_O_2_ Eq/L.

The division of TOS by TAS yields the oxidative stress index (OSI) value. The following formula is used to calculate OSI: OSI (arbitrary unit)=TOS (µmol H_2_O_2_ Eq/L)/TAS (mmol Trolox Eq/L)^(17)^.

The analysis of chromosomes was conducted in the Department of Medical Biology-Genetics. Blood samples were taken into heparinized vacutainers for cytogenetic analysis, and the lymphocyte cultures were organized in duplicates^(18)^. Two sets of slides were arranged from each culture. Karyotyping was conducted on routine peripheral blood lymphocyte cultures using G-banding following Trypsin and Giemsa staining (GTG)^(19)^. A minimum of 30 GTG-banded metaphases were scored from each patient. Considering the criteria of the International System for Human Cytogenetic Nomenclature, three cells were karyotyped^(20)^. In general, chromosome counts of 30 cells were undertaken; however, 50 or more cell counts were performed in the event that mosaicism was suspected^(21)^.

### Statistical Analysis

Statistical Package for the Social Science (SPSS 21) (IBM SPSS Version 8.5.0.0021 Licensed Materials Property of IBM Corp. Copyright IBM Corporation and others) was used for data analyses. Data analyses are expressed as mean and standard deviation. Student’s t-test and Mann-Whitney U test were used to compare clinical and biochemical data between the groups. Whether intra-group variables demonstrated normal distribution was determined using the Kolmogorov-Smirnov test. The investigation of correlation between the values was conducted using Spearman’s analysis. P values less than or equal to 0.05 were considered statistically significant.

## RESULTS

**Figure 1:**
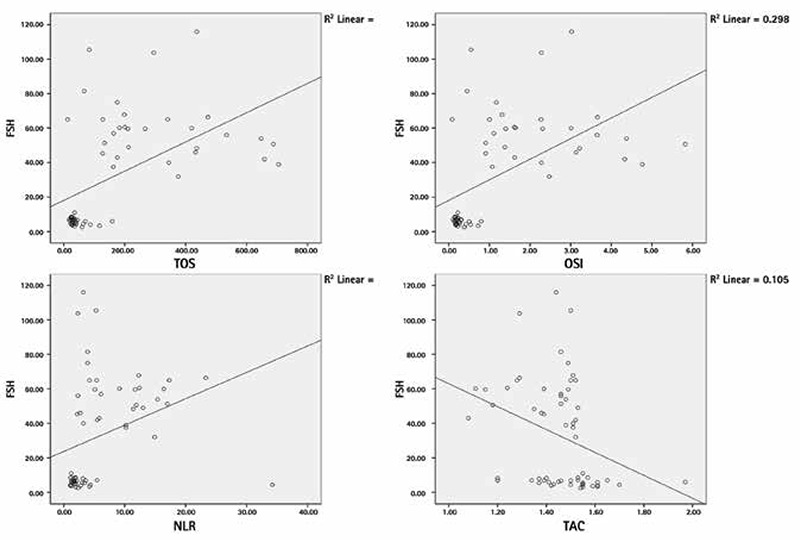
Correlation analysis between follicle-stimulating hormone, total antioxidative capacity, total oxidative capacity, oxidative stress index, and neutrophil to lymphocyte ratio *NLR: Neutrophil to lymphocyte ratio, OSI: Oxidative stress index, FSH: Follicle-stimulating hormone, TOS: Total oxidant status*

**Figure 2:**
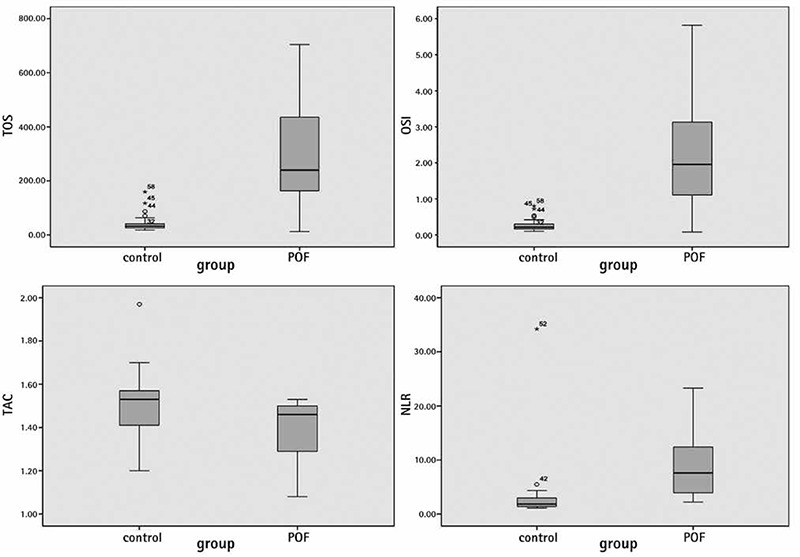
Neutrophil to lymphocyte ratio, total antioxidative capacity, total oxidative capacity, oxidative stress index levels in patients with premature ovarian insufficiency and controls *POF: Premature ovarian failure, TOS: Total oxidant status*

No participants in this study had a pathologic presentation on pelvic ultrasound or a chromosomal abnormality. In addition, none of the participants had a family history of POI. No significant differences were found between the patients with POI and control subjects in age, marital status, gravidity, smoking and BMI (Table 1). No significant differences were found between the groups in lipid profile levels, TSH, PRL, or glucose. E2 levels were lower in patients with POI than in the control subjects p<0.001 (Table 2). However, NLR and levels of FSH, LH, TOS, and OSI were higher in patients with POI than in the control subjects (Table 3) (Figure 1). The levels of FSH were directly correlated with TOS, OSI, and NLR (r=0.573** p<0.001; r=0.584** p<0.001; r=0.541 p<0.001, respectively) and indirectly correlated with TAS (r=-0.437** p<0.001) (Figure 2).

## DISCUSSION

In our study, we hypothesized that there would be increased OS, and investigated the presence of inflammation in patients with POF. Many studies have been conducted on ovarian reserve and OS. However, there are few studies in patients with POF. We determined increased OS and inflammation in patients with POF.

Various etiologic factors are associated with POI: Autoimmune ovarian damage, genetic aberrations, infectious agents, toxins, iatrogenic factors, and environmental factors. Nevertheless, the majority of cases are idiopathic with no identifiable etiologic factors, even after a thorough examination^(22)^.

POI is usually sporadic; however, one of the first-degree relatives of 10-15% of patients also has this disorder^(23)^. Therefore, patients should be asked about their family history. In addition, hypothyroidism, adrenal insufficiency, hypoparathyroidism, and other autoimmune diseases should be questioned. A family history of mental retardation, tremor-ataxia, and Parkinson-like symptoms might lead to consider Fragile X syndrome associated with FMR1 gene mutation^(24)^. However, no patients with POI in the present study had a family history or an autoimmune disease.

Chronic low-grade inflammation is a major contributor to the pathogenesis of POI. NLR, which is an indicator of systemic inflammation, demonstrates the balance between neutrophil and lymphocyte concentrations in the body. Compared with many other inflammatory markers, NLR adds no extra cost because it is inexpensive. It is widely available and routinely measured on admission. In the present study, NLR was found to be significantly elevated in patients with POI, which might indicate the potential role of increased inflammation in the etiology of POI. Previous studies reported that patients with POI had an increased risk of atherosclerosis and CVD^(25)^ which might also be associated with increased inflammation in these patients.

The effect of OS in the etiopathogenesis of POF has not been widely studied. A recent study reported that administration of coenzyme Q in patients with POF who had high reactive oxygen species (ROS) levels improved the embryo quality^(26)^. High superoxide ion amounts lead to a decline in the bioavailability of nitric oxide, an accrete in ROS levels, and OS. As compared with spermatozoa, female germ cells improve under hypoxic conditions in the ovarian cortex. However, exposure to supraphysiologic levels of ROS are deleterious to developing oogonia. Another study proposed that increased production of OS contributed to oophoritis associated with POF^(27)^. High ROS levels stimulate mitochondrial DNA modifications. In addition, high ROS levels lead to mitochondria abnormality, which could lead to low adenosine triphosphate production due to disrupted oxidative phosphorylation and oogenesis, low oocyte number, and POF^(28)^.

OS is involved in the etiology of diverse degenerative conditions such as diabetes, atherosclerosis, arthritis, cancer, and aging^(29)^. In addition, it was shown that ROS such as hydroxyl radicals, hydrogen peroxide, and superoxide anions are a part of the pathogenesis of bone loss caused by osteoclast differentiation and bone resorption^(30)^. Furthermore, two previous studies suggested that ROS levels were elevated in POI, and OS may be a part of the etiology of idiopathic POI^(31,32)^.

The role of OS in female fertility is an area worthy of sustained research. Preliminary research that analyzes the complete mitochondrial genome and OS should be tracked in different populations and in broader studies. This research should also investigate the ovary for mitochondrial nucleotide change because they develop in a different microenvironment in the ovary and are of different embryologic origin. However, owing to ethical constraints, such research is not feasible. In a study of male infertility from our laboratory, we found systemic blood ROS levels correlated with seminal ROS levels^(33)^. Hence, the present study on blood TAS, TOS, and OSI in POI is significant, although it would be ideal to conduct further, similar studies on oocytes. The therapeutic tools currently available for the treatment of mitochondrial diseases due to mitochondrial deoxyribonucleic acid mutations are very few, and their efficacy is not yet well established^(34)^.

In a large series of 357 patients with POI, the median E2 level was only 10 pg/mL^(35)^. In the present study, estrogen levels were found significantly lower in patients with POI compared with the control subjects.

There is no family history in the vast majority of women with POI. The chromosomes are normal, and there is no sign of auto-immunity in these women, which renders the mechanism of damage unknown. Therefore, hidden environmental damage from the past might be considered in these women. In men, it is known that viruses such as mumps might cause testicular inflammation and lead to permanent damage and lack of sperm. In addition, it is widely believed that sperm counts in men have reduced in recent years because of testicular exposure to environmental toxins and drugs. In this respect, it is probable that the ovaries are similarly affected by viruses and toxins. Viruses in particular are a potential cause of ovarian insufficiency in women with no identifiable cause. Anecdotal reports of virus infections that are rapidly followed by ovarian insufficiency provide support for this causal relationship(35). In the present study, NLR was found significantly elevated in patients with POI who had no history of inflammation or medication. As a result, idiopathic POI might be explained by inflammation caused by environmental toxins. Smoking is known to be detrimental to ovarian functions. On average, smokers experience menopause earlier than nonsmokers, which indicates a potential harmful effect of smoking on ovarian functions^(36)^. Our study demonstrated no significant difference between patients with POI and healthy control subjects regarding smoking.

Previous studies reported that a diet rich in glucose and free fatty acid could trigger OS and an inflammatory response from mononuclear cells^(37)^. The present study found no significant difference between patients with POI and controls in lipid profile and glucose levels. Therefore, increased OS observed in patients with POI might be associated with increased inflammation.

### Study Limitations

A limitation of our study is that we did not measure anti-Müllerian hormone (AMH) levels. The AMH test is the best to evaluate the ovarian reserve, but it cannot be performed in our hospital; instead ovarian reserve was evaluated through FSH, LH, and E2 measurements. FSH and E2 are important markers of ovarian reserve, especially in the absence of known AMH levels.

As far as we know, no other studies have investigated NLR, TAS, TOS, and OSI in patients with POI. The present study revealed elevated levels of NLR, TOS, and OSI in patients POI. Therefore, anti-oxidative and anti-inflammatory treatment might be administered to patients in the early stage of POI. However, larger studies are needed to clarify whether these elevated levels are a cause or a consequence of POI.

## Figures and Tables

**Table 1:**
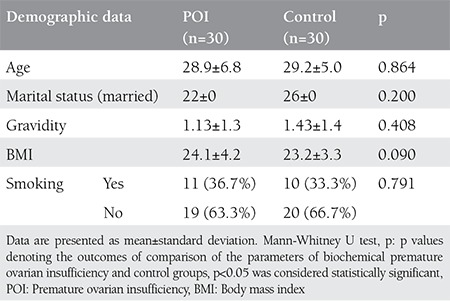
Demographic data of the groups

**Table 2:**
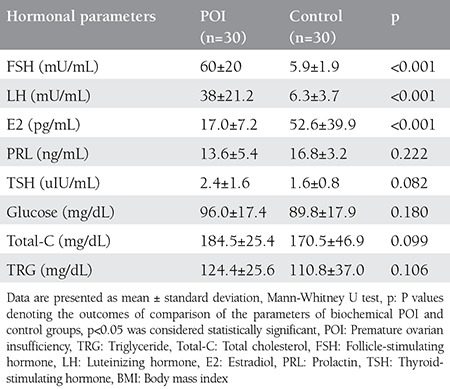
Levels of hormonal parameters in the groups

**Table 3:**
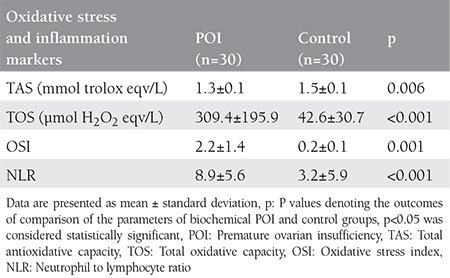
Levels of oxidative stress and inflammation markers in the groups
